# Supplementary material for: Genome-Wide Identification and Expression Patterns of the SWEET Gene Family in Bletilla striata and its Responses to Low Temperature and Oxidative Stress
Source: Int J Mol Sci. 2022 Sep 2;23(17):10057. doi: 10.3390/ijms231710057 (PMC9456286; doi:10.3390/ijms231710057)
Supplement: Supplementary file 1 [file ijms-23-10057-s001.zip › ijms-1872815-supplementary.pdf]

Table S1. Information of *BsSWEET* genes.

Gene name	Chromosome location	Protein(aa)	MW(Da)	Theoretical pI	Aliphatic index	GRAVY	Subcellular localization
BsSWEET1	Chr1	262	28,718.14	9.50	107.67	0.571	Plasma membrane
BsSWEET2	Chr1	213	23,440.78	9.17	115.77	0.408	Plasma membrane
BsSWEET3	Chr2	258	28,578.46	8.70	136.59	0.938	Plasma membrane
BsSWEET4	Chr3	237	26,845.94	8.67	118.10	0.903	Plasma membrane
BsSWEET5	Chr4	190	21,349.94	5.85	135.89	1.058	Plasma membrane
BsSWEET6	Chr4	162	18,209.80	8.52	126.85	0.610	Plasma membrane
BsSWEET7	Chr4	261	29,873.61	9.48	114.60	0.498	Plasma membrane
BsSWEET8	Chr4	198	22,355.01	9.32	132.88	0.897	Plasma membrane
BsSWEET9	Chr4	112	12,497.44	5.47	95.00	0.521	Plasma membrane
BsSWEET10	Chr4	250	27,479.81	9.68	129.00	0.681	Plasma membrane
BsSWEET11	Chr5	259	28,872.71	8.48	130.54	0.850	Plasma membrane
BsSWEET12	Chr5	113	12,621.89	7.82	114.69	0.347	Plasma membrane
BsSWEET13	Chr6	142	16,132.84	8.96	152.18	1.363	Plasma membrane
BsSWEET14	Chr7	236	26,222.60	8.78	120.51	0.790	Plasma membrane
BsSWEET15	Chr7	247	28,195.57	9.01	104.09	0.527	Plasma membrane
BsSWEET16	Chr8	299	33,984.56	9.31	116.35	0.629	Plasma membrane
BsSWEET17	Chr8	269	29,659.77	7.00	114.94	0.401	Plasma membrane
BsSWEET18	Chr9	220	24,559.24	9.18	117.45	0.828	Plasma membrane
BsSWEET19	Chr10	176	19,896.92	9.75	132.33	0.757	Plasma membrane
BsSWEET20	Chr11	258	29,125.70	9.42	110.74	0.436	Plasma membrane
BsSWEET21	Chr11	269	30,202.80	7.66	120.37	0.694	Plasma membrane
BsSWEET22	CTG2248	241	27,957.82	9.63	122.45	0.572	Plasma membrane
BsSWEET23	CTG4490	258	28,586.48	8.70	132.44	0.903	Plasma membrane

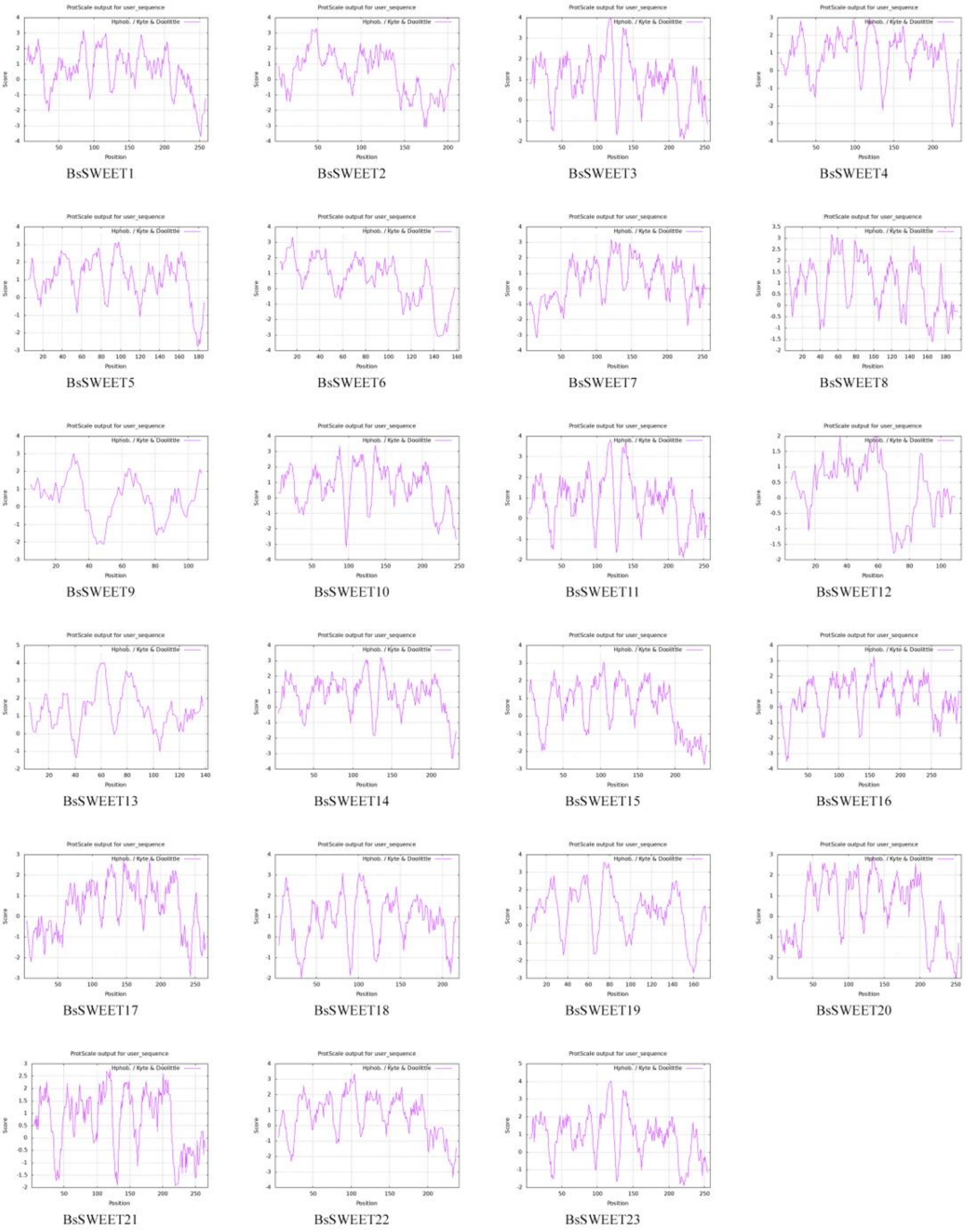

Figure S1. Prediction of hydrophobicity of BsSWEET proteins.

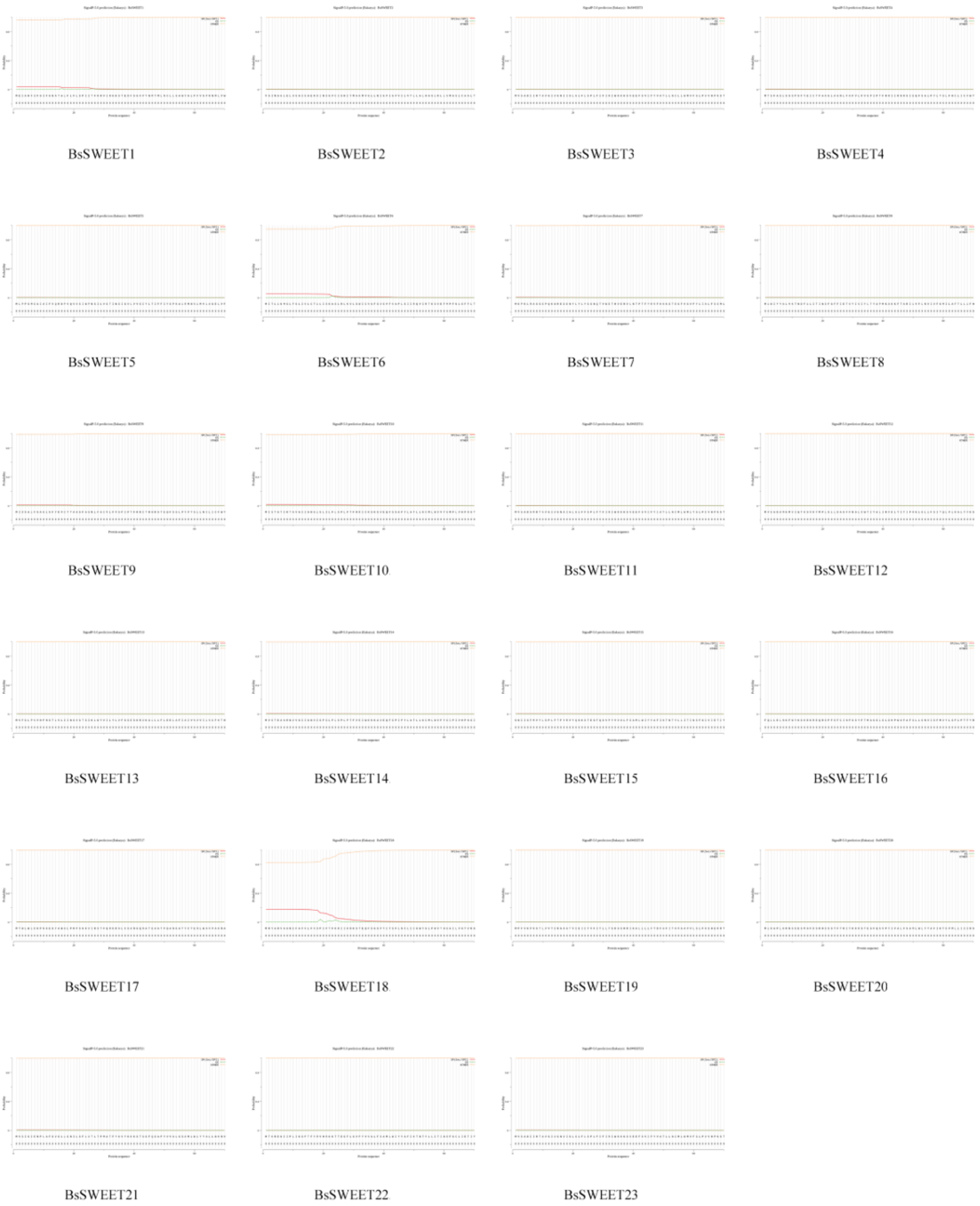

Figure S2. Prediction of signal peptides of BsSWEET proteins.

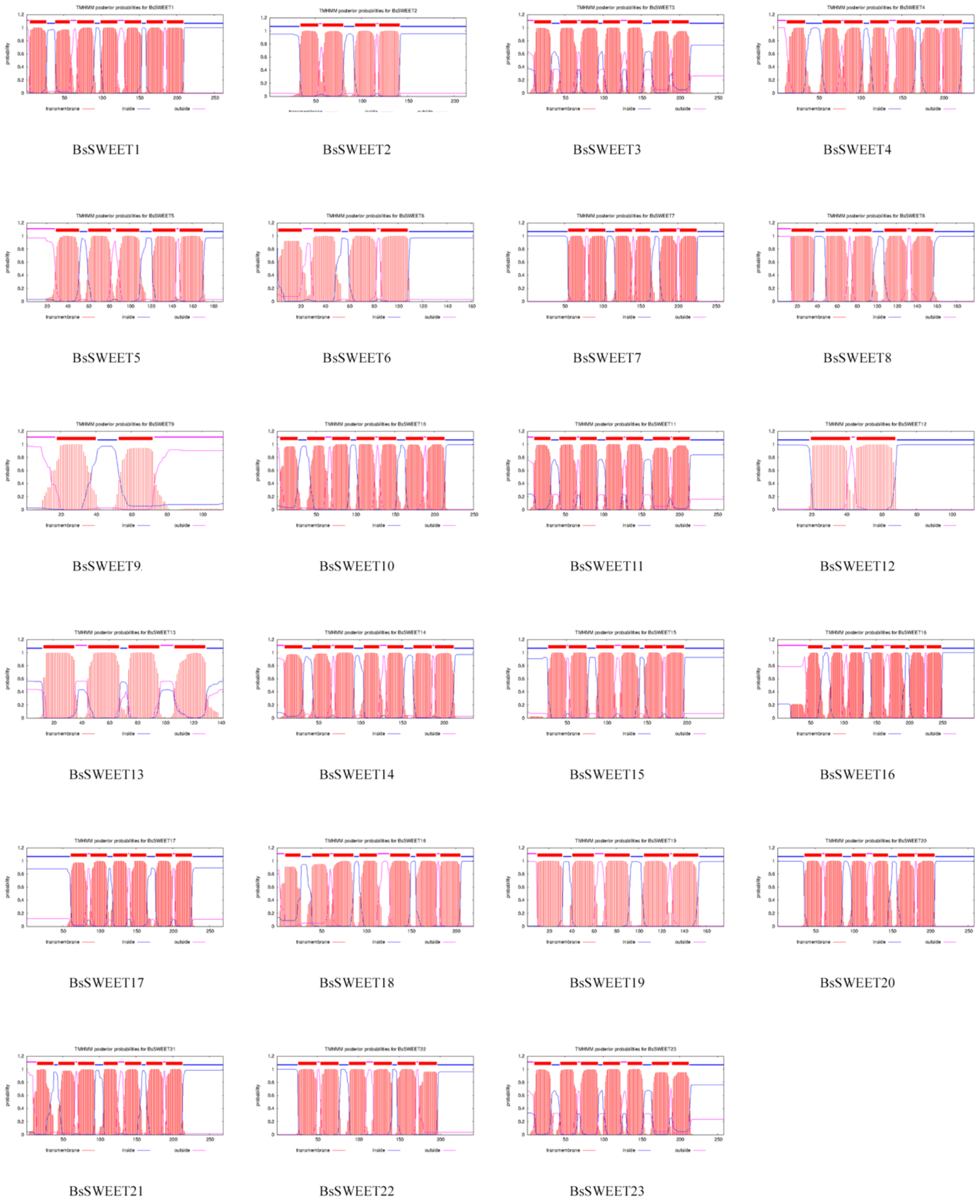

Figure S3. Prediction of the transmembrane structural domain of BsSWEET proteins.

Table S2. Prediction of the secondary structure of BsSWEET proteins.

	Alpha helix	Beta turn	Random coil
BsSWEET1	42.37%	3.82%	33.21%
BsSWEET2	41.78%	5.63%	31.92%
BsSWEET3	37.98%	3.88%	36.43%
BsSWEET4	44.73%	2.95%	31.22%
BsSWEET5	40.53%	7.89%	23.16%
BsSWEET6	37.04%	1.23%	40.12%
BsSWEET7	39.46%	2.30%	38.31%
BsSWEET8	42.93%	2.02%	30.81%
BsSWEET9	41.07%	3.57%	3.57%
BsSWEET10	38.40%	4.80%	36.40%
BsSWEET11	39.38%	3.47%	36.29%
BsSWEET12	37.17%	1.77%	36.28%
BsSWEET13	39.44%	4.23%	26.76%
BsSWEET14	44.92%	3.39%	28.81%
BsSWEET15	39.68%	3.24%	36.44%
BsSWEET16	40.80%	3.34%	35.12%
BsSWEET17	38.29%	5.58%	34.20%
BsSWEET18	39.55%	2.73%	33.18%
BsSWEET19	40.91%	4.55%	28.98%
BsSWEET20	38.76%	3.88%	37.21%
BsSWEET21	46.84%	1.49%	34.20%
BsSWEET22	45.23%	3.32%	31.12%
BsSWEET23	42.64%	3.10%	33.33%

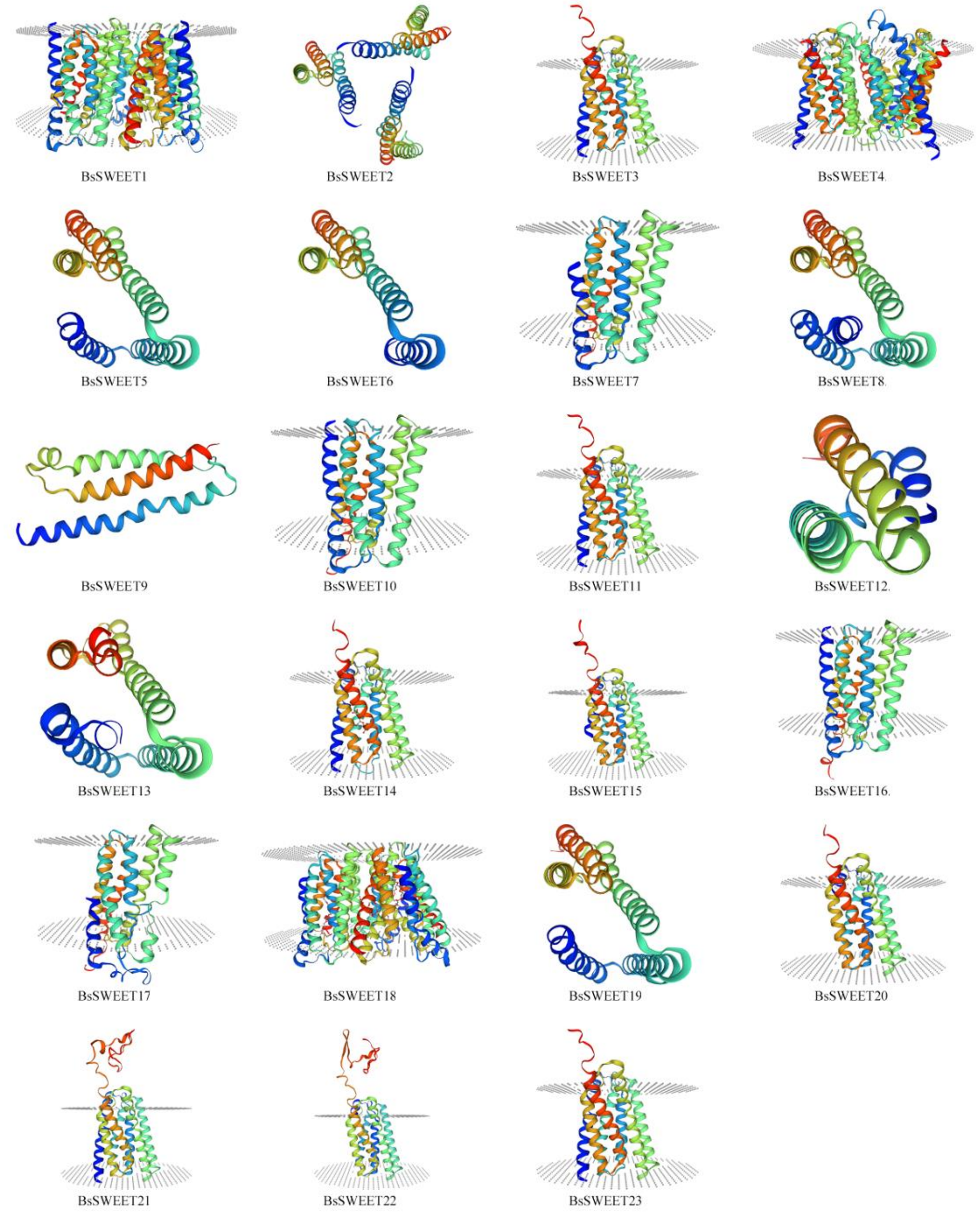

Figure S4. Prediction of the tertiary structure of BsSWEET proteins.

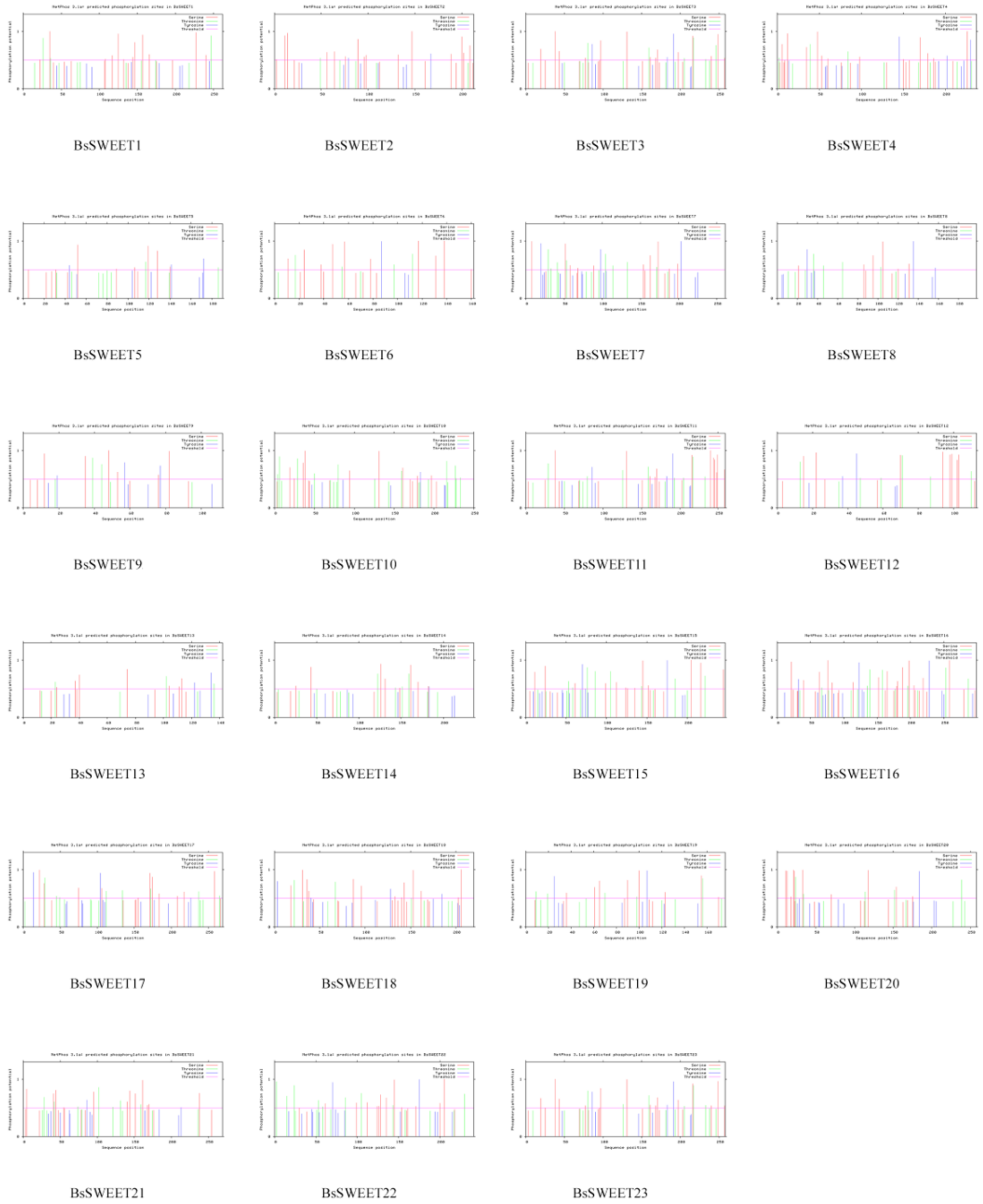

Figure S5. Prediction of phosphorylation sites of BsSWEET proteins.

Table S3. Prediction of phosphorylation sites of BsSWEET proteins.

	Ser site	Thr site	Tyr site
BsSWEET1	12	4	0
BsSWEET2	16	2	2
BsSWEET3	15	6	3
BsSWEET4	14	3	3
BsSWEET5	8	2	3
BsSWEET6	10	4	1
BsSWEET7	10	8	3
BsSWEET8	6	4	3
BsSWEET9	6	3	3
BsSWEET10	10	12	1
BsSWEET11	12	8	3
BsSWEET12	8	6	2
BsSWEET13	7	3	2
BsSWEET14	8	4	1
BsSWEET15	13	8	3
BsSWEET16	15	8	3
BsSWEET17	12	8	3
BsSWEET18	13	3	4
BsSWEET19	9	7	2
BsSWEET20	10	5	1
BsSWEET21	12	7	2
BsSWEET22	13	9	2
BsSWEET23	15	6	3

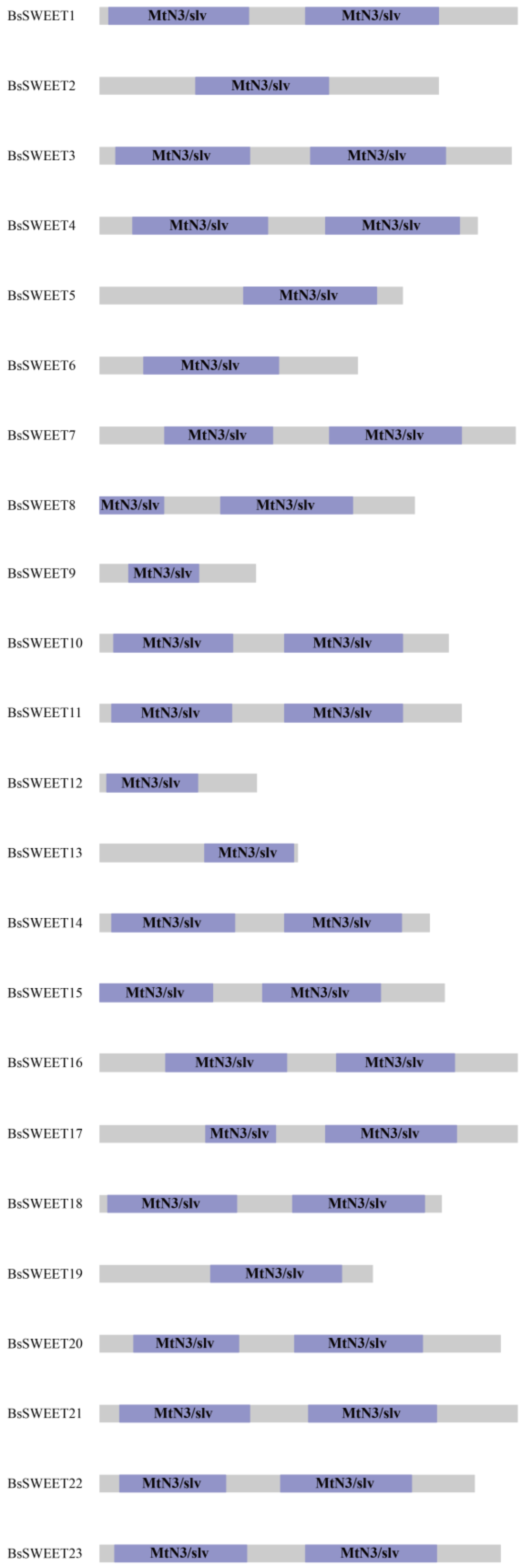

Figure S6. The SWEET conserved domains in *B. striata*.

Table S4. The sequence of the conserved motifs in *B. striata*.

		MEME sites
motif 1	HSRRLLIVGIJCVIFSVSMYAAPLSIMRLVIRTKSVEFMPF	21
motif 2	FLFLNGVCWTIYGLJSKDIYITJPNGLGLLFGIAQLLLYAIYKKSKKPQ	21
motif 3	PLPTFYRIYKKKSTEQFSSVPYVVALLNCMLWIYYGLPKVH	15
motif 4	LVLTINSFGTVIETIYIIJYLIYAPKKKR	19
motif 5	EKEMKGEVGLTDVVIDDSVKPPPTVASHK	4
motif 6	DIIRFAVGIVGNVISLGLFLS	10
motif 7	FTAKIILLLNVGIFGAIVLCT	8
motif 8	FLGELAFVAIVVVVVLVCFHT	7

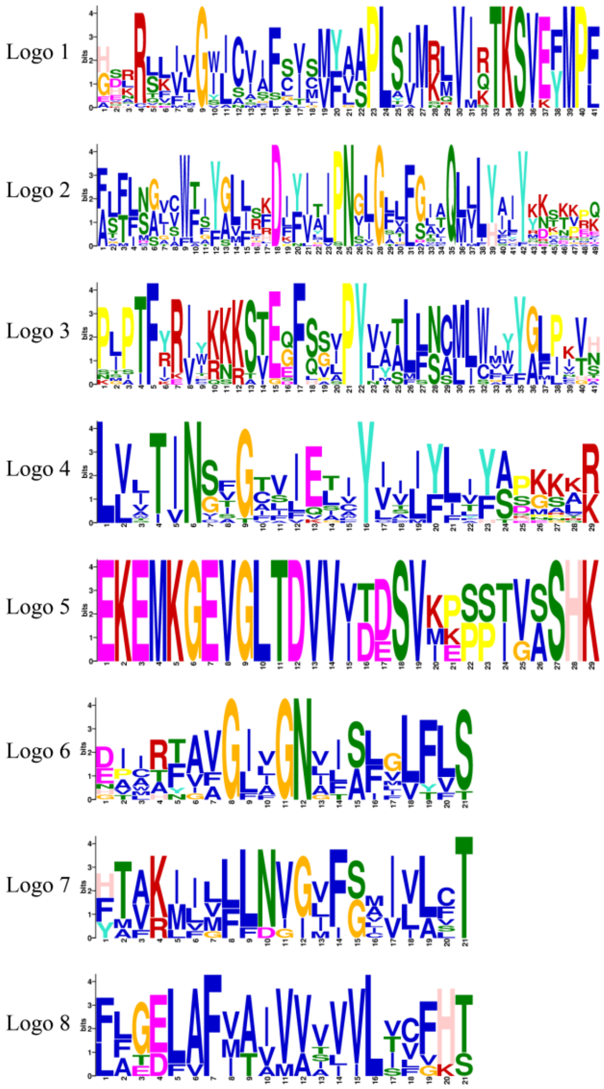

Figure S7. The different conserved motifs in *B. striata*. The letter size of the amino acid represents the frequency of the corresponding nucleotide.

Table S5. Detailed information on *cis*-acting elements of the promoter region in *B. striata*.

cis--acting element	sequence	response
G-box	GCCACGTGGA	Sugar suppress
G-box	CACGTG	Sugar suppress
I-box	AGATAAGG	Sugar suppress
I-box	TGATAATGT	Sugar suppress
I-box	AGATAAGG	Sugar suppress
W box	TTGACC	Sugar induce
ABRE	ACGTG	abscisic acid responsiveness
ACE	CTAACGTATT	light responsiveness
CAT-box	GCCACT	related to meristem expression
CGTCA-motif	CGTCA	MeJA responsiveness
GARE-motif	TCTGTTG	gibberellin responsiveness
LTR	CCGAAA	low-temperature responsiveness
P-box	CCTTTTG	gibberellin responsiveness
TCA-element	CCATCTTTTT	salicylic acid responsiveness
TC-rich repeats	ATTCTCTAAC	defense and stress responsiveness
TCT-motif	TCTTAC	light responsiveness

Table S6. Parameter estimates and log-likelihood scores under models of ω-ratios among sites.

Model	parameter estimates	Likelihood scores	Positive selected sites
M0:One-ratio	ω_0=0.10954	-207.827844	Not Allowed
M1a:Nearly neutral	ω_0=0.02710, ω_1=1, (p_0=0.82381, p_1=0.17619)	-197.067484	Not Allowed
M2a:Positive selection	ω_0=0.02710, ω_1=1, ω_2=1,(p_0=0.82381, p_1=0.04344, p_2=0.13275)	-197.067485	[]
M3:discrete	ω_0=0.02710, ω_1=0.02098, ω_2=0.52812, (p_0=0, p_1=0.82072, p_2=0.17928)	-196.359044	[]
M7: β	p=0.48953, q=1.41328	-195.873654	Not Allowed
M8:β+ωs>1	p_0=0.84124, p_1=0.15876, p=1.02856, q=11.54106, ω=1	-196.511480	
M8a:β+ωs=1	p_0=0.83485, p_1=0.16515, p=1.45584, q=122.48221, ω=1	-196.877938	Not Allowed

Table S7. List of q-PCR validation primers used in the present study.

Gene ID	Forward primer (5′ - 3′)	Reverse primer (5′ - 3′)
BsSWEET1	ACGGGTTGCTTGGCCATGAT	CGCCCATCTCAACGGCTTCT
BsSWEET2	TGCAGGGATTTGGACAATCTACTCTGT	CTGAGCTGAGCCTTCTTCTTCTGC
BsSWEET3	GGGTCAAAGCGGGTTAAGGCA	GGAGCGACGATCGTGTGTGT
BsSWEET4	CGTCCGGTCCCTCTTTCTAT	AGCAAACAGGTTTCCTGCAA
BsSWEET5	GGTCACTTTGGTGCTCACGGT	AGGCAGCGAAGGAAAGCCAA
BsSWEET6	AGCGCAGTGGTTTGGTTCTCC	TGAAGCGCACCAAATGTGAATCCT
BsSWEET7	GCGTTGCGTTCTCTGTGAGC	ACAGTACACCGTAGCCTAGCCA
BsSWEET8	CGTTTGGCTAGGCTACGGTGT	TCAGCAAGCTTCGGATTGGCA
BsSWEET9	TTTCGGCCGGCCTTTCTTTCT	GGCGACACGAAGAGTACGATGG
BsSWEET10	TCCACTCGCAAGGCAAGCAA	ACAAGCCCAACAGCAGCGAT
BsSWEET11	TGTGCTGGGACAGCCATTGAG	AGAAGAGCAGCATGGCCTTGAG
BsSWEET12	GGTTTCTGCCGATGCAATGA	AGGCCAAGACCATTGGGAAT
BsSWEET13	TGGTGTTTGGGCTACCCGTTG	GCCTTAACCCGCTTCGACTCA
BsSWEET14	GCCCGAAACGTCGTTGGGAT	CGGGATTGGCGAGAACTGCT
BsSWEET15	TCTTCCAAGGCTCTAATCGCCTCA	CACTCTTGGTGCGGATGACCA
BsSWEET16	CTGAAAGCTTGGCAGAGGAGCA	GACAACCTGTGGCTTGGTCACT
BsSWEET17	AAGCAGCTACACCGCAAGCA	GATGCGCCGAGGAGAGCATT
BsSWEET18	AGAAGGCAAAGCTCGGCAGAAT	TTGCAAGAGAAAGGTGGAAAGGCA
BsSWEET19	ACCAAACAGCACACTTGTGGTCA	GGCGAAGTCGGTGAAGAGCA
BsSWEET20	GGGTTTCAGGCGGTGCCTTAT	AACGCAGCCGATGGAGTTGAT
BsSWEET21	GGTGGCTTTGTCCAGTGCGA	AGGCAGATTCAAGGACGCAAGTG
BsSWEET22	TCAGTTAGTGTCTTCGCTGCTCCT	TGCAAATCCAAGCACATTTGGGAGT
BsSWEET23	GGTCGAAGCGGGTTAAGGCA	ATGCCGACGATGAGGGAACG
BsGAPDH	GCTAAAGACGCTGTCACTGAG	GAGACCAGAAACTTACCCTCG

Table S8. List of primers used in the present study.

Gene ID	Forward primer (5′ - 3′)	Reverse primer (5′ - 3′)
BsSWEET 15	GGGGTACCGGAAATATCATCTCATTCATGGTGTACCT	CGGGATCCCACCGGGCTCATCTCCTTC
BsSWEET 16	GGGGTACCTTCCAATTACTTGAGTTAAACAAGTTTTGG	CGGGATCCCACTGCGCTTATCTCATTGTA
